# ^1^H, ^13^C, and ^15^N backbone and side-chain resonance assignments of the *Nostoc sp.* H-NOX C139A variant in complex with the soluble Guanylyl Cyclase inhibitor Zinc Protoporphyrin IX

**DOI:** 10.1007/s12104-026-10274-5

**Published:** 2026-08-26

**Authors:** Stefanos I. Gravalos, Emmanouil N. Andreadis, Francesca Cantini, Lucia Banci, Georgios A. Spyroulias

**Affiliations:** 1 Department of Pharmacy, University of Patras, 26504 Patras, Greece; 2 Magnetic Resonance Center - CERM, University of Florence, Via Luigi Sacconi 6, Sesto Fiorentino, 50019 Florence, Italy; 3 Department of Chemistry, University of Florence, Via della Lastruccia 3, Sesto Fiorentino, 50019 Florence, Italy

**Keywords:** H-NOX, Soluble Guanylyl Cyclase, sGC, NMR spectroscopy, *Nostoc sp.*, Inhibitor

## Abstract

The H-NOX (Heme–Nitric oxide/Oxygen binding) domain is highly conserved among both eukaryotes and bacteria. In human soluble Guanylyl Cyclase (sGC), the H-NOX domain functions as a sensor for the gaseous signaling molecule Nitric Oxide (NO). Located at the N-terminus of the enzyme, the heme B-binding H-NOX domain regulates the catalytic domain at the C-terminus, which catalyzes the conversion of GTP (guanosine 5′-triphosphate) to cGMP (cyclic Guanosine MonoPhosphate). The present study employs the bacterial homolog *Nostoc sp.* H-NOX (*Ns* H-NOX) C139A mutant together with the sGC inhibitor Zinc Protoporphyrin IX (ZnPPIX) as experimental tools to investigate the still poorly understood mechanisms underlying sGC activation and inhibition, and to elucidate either the chemical features of novel scaffolds associated with a new class of activators, or the distinct activities imposed by two prosthetic groups sharing the same organic framework, but differing in the chelated transition metal. Here, we present the conditions employed for the formation of the complex between the *Ns* H-NOX C139A mutant and the sGC inhibitor ZnPPIX, together with the UV–Vis absorption spectra of the heme-bound and ZnPPIX-bound protein forms. Furthermore, we report the NMR backbone and side-chain resonance assignments (^1^H, ^13^C, ^15^N) of the complex, as well as the chemical shift-based secondary structure predictions. These data will be used for comparative analysis of ^15^N NMR relaxation experiments in the basal, fully active and inactive H-NOX states, aiming to elucidate the role of Η-ΝΟΧ protein dynamics in the initiation and propagation of sGC activation signal.

## Biological context

Soluble guanylyl cyclase (sGC) is a vital enzyme in the NO/cGMP signaling pathway, with its disfunction being related with various pathophysiological conditions including cardiopulmonary (Stasch et al. 2011), renal (Krishnan et al. 2018) and neurological diseases (Correia et al. 2021). The enzyme’s implication in these conditions has made it a highly relevant therapeutic target, rendering the elucidation of its activation mechanism of the utmost importance.

sGC is a heterodimeric enzyme, with the most prominent isoform consisting of an *α1* and a *β1* subunit. The *β1* subunit bears a heme cofactor in its N-terminal, regulatory Heme Nitric oxide/Oxygen binding (H-NOX) domain, bound by formation of a coordination bond between the heme iron and a histidine residue. During enzyme activation, NO acts as a regulatory molecule, binding on the heme iron and causing cleavage of the iron-histidine bond (Cary et al. 2006; Zhao et al., n.d.). NO coordination on heme iron is believed to be the initial step in enzyme activation, inducing conformational changes eventually transduced to the C-terminal, catalytic region and allowing for the conversion of guanosine 5-triphosphate (GTP) to cyclic guanosine 3,5-monophosphate (cGMP) (Underbakke et al. 2014).

**Fig. 1:**
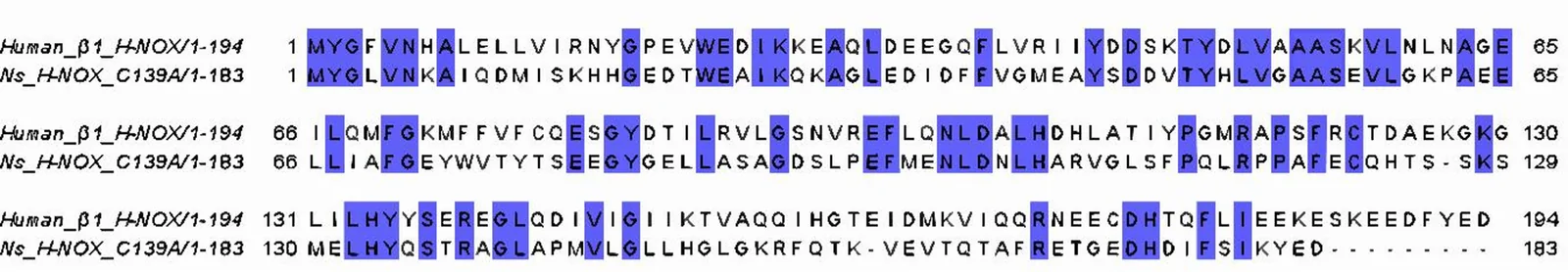
Sequence alignment of the human sGC *β1* H-NOX domain and the *Nostoc sp.* H-NOX protein. Residue numbering is based on the sequence of the human *β1* H-NOX domain. Conserved residues between the two proteins are highlighted in blue

The most rigorously studied part of the enzyme has, naturally, been the point of signal initialization, the H-NOX domain. Prokaryotic homologues of the *β1* H-NOX domain have been widely utilized in structural studies and functional assays to identify key residues that participate in gas discrimination and ligand binding (Boon and Marletta 2005). In particular, the H-NOX protein that is expressed in the cyanobacterium *Nostoc sp.* (*Ns* H-NOX) has been used extensively due to its high sequence identity to human sGC’s *β1* H-NOX domain (33.86%) (Fig. 1) and the fact that 17 of the 27 residues in the heme pocket are identical between the two proteins (Ma et al. 2007).

Zinc protoporphyrin IX (ZnPPIX) is a high affinity ligand for the *β1* heme-binding pocket of sGC and has long acted as a key tool for studying enzyme inhibition. It has been shown to decrease basal activity and inhibit NO induced activation in both heme-depleted and native enzyme forms (Ignarro et al. 1984; Serfass and Burstyn 1998), while also exhibiting inhibition that is competitive to sGC activators such as 4-[((4-carboxybutyl){2-[(4-pheneth ylbenzyl)oxy]phenethyl}amino) methyl [benzoic]acid (BAY 58-2667, Cinaciguat) (Stasch et al. 2006). Importantly, ZnPPIX also appears to prevent oxidation induced proteasomal degradation, in conditions where the heme deficient form of the enzyme is likely to be prevalent (Hoffmann et al. 2009; Stasch et al. 2006). Nevertheless, no structural data has yet been provided that directly indicates the occupation of the heme-binding pocket by ZnPPIX.

In previous studies our group has published the backbone and sidechain NMR resonance assignments of the *Ns* H-NOX C139A mutant, in its free state (Alexandropoulos et al. 2016) as well as in complex with Cinaciguat (Makrynitsa et al. 2021). The present study reports the backbone and sidechain NMR resonance assignment of *Ns* H-NOX C139A in complex with ZnPPIX, providing for the first time experimental data for the replacement of the heme prosthetic group by the ZnPPIX. This work, in conjunction with our previous findings, allows us to study and compare *Ns* H-NOX in three distinct conditions simulating the inactive, fully active and, presently, inhibited form of sGC, by solution NMR. This in turn assists in further understanding of the complete activity profile of sGC and the conformational and dynamic changes that accompany the transition between these different states, consequently shedding light on the enzyme’s activation mechanism.

## Methods and experiments

### Protein expression and purification

**Fig. 2:**
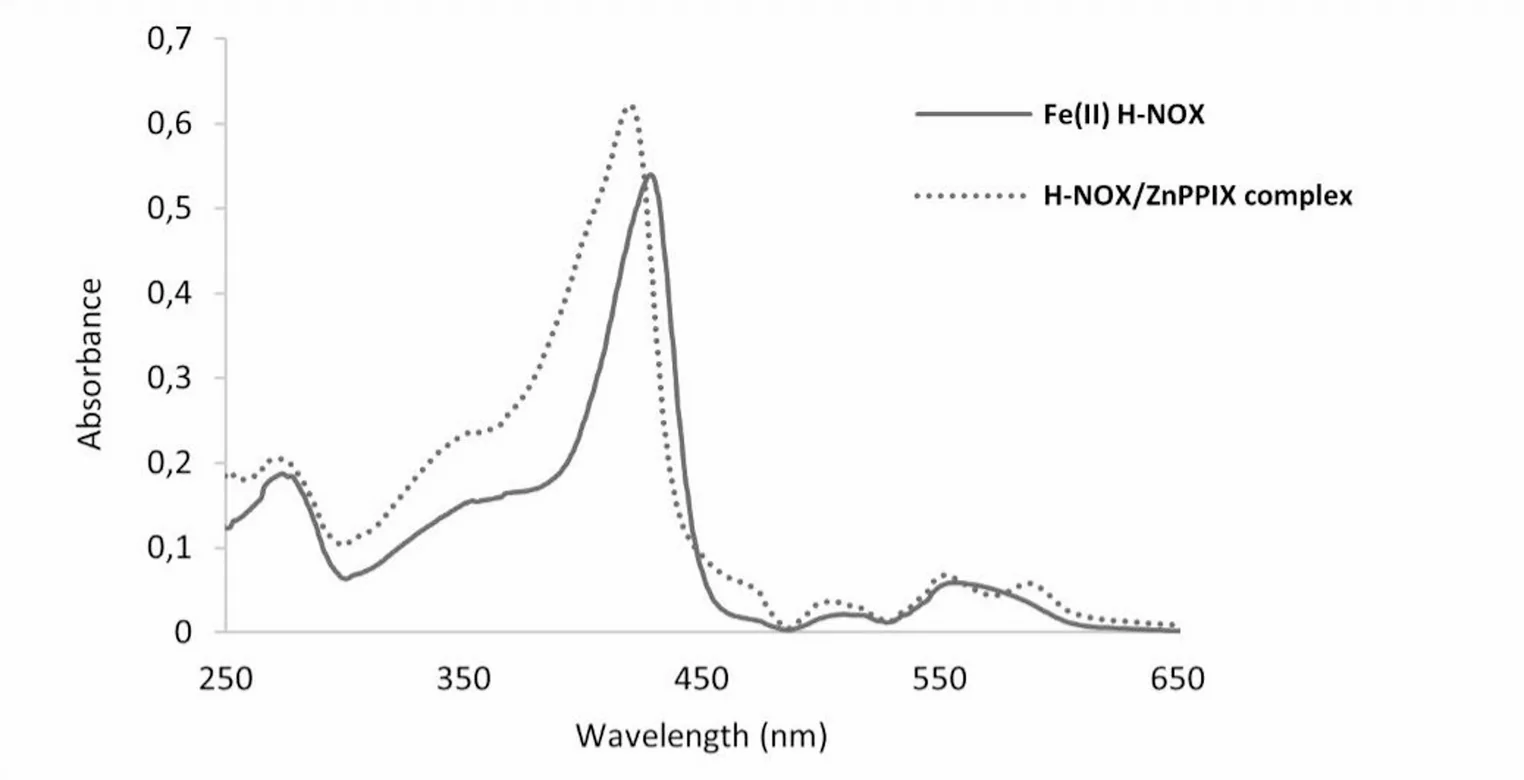
UV–Vis absorption spectra of the heme in the *Ns* H-NOX C139A mutant (solid line) and of the mutant in complex with ZnPPIX (dashed line). The heme exhibits a Soret-band absorption maximum at 429 nm, indicating that the iron is in the oxidation state Fe(II). Following replacement of the heme with ZnPPIX in the *Ns* H-NOX C139A mutant, the Soret band displays an absorption maximum at 421 nm

The gene encoding the H-NOX C139A mutant of *Nostoc sp.* (*Ns* H-NOX) was cloned into pET22b(+) expression vector and was kindly provided by Dr Focco van den Akker (Case Western Reserve University, USA). The H-NOX protein, comprising residues 1-183 of the *Ns* H-NOX domain, was expressed in *Escherichia coli* BL21 (DE3) Star cells. Cells were grown at 37 °C in M9 minimal medium supplemented with ^13^C (4 g/L) and ^15^N isotopes (1 g/L) and 0.3 mM δ-aminolevulinic acid (ALA) to promote heme biosynthesis until an OD600 of approximately 0.9 was reached. The temperature was then reduced to 18 °C, and protein expression was induced by the addition of 0.2 mM isopropyl β-D-thiogalactopyranoside (IPTG). Purification of *Ns* H-NOX C139A mutant was carried out as previously described (Alexandropoulos et al. 2016). The UV–Vis absorption spectrum of the *Ns* H-NOX C139A mutant exhibits a Soret band at 429 nm, consistent with a five-coordinate Fe(II) heme complex (Fig. 2). Together with previous reports on bacterial H-NOX proteins, these findings strongly indicate that the *Ns* H-NOX C139A mutant exists predominantly in the diamagnetic Fe(II)–H-NOX complex form (Tsai et al. 2010).

### NMR sample preparation for the *Ns* H-NOX/ZnPPIX complex

The native heme was replaced with Zinc protoporphyrin IX (ZnPPIX) by adding a 4-fold molar excess of the highly selective sGC inhibitor and heme oxidizing agent ODQ (Sigma 495320), together with 4-fold molar excess of ZnPPIX (Cayman 14483). Both compounds were initially dissolved in DMSO-*d*_*6*_ (Eurisotop D010B). Following successful formation of the complex, a PD-10 desalting column was used to remove the DMSO-*d*_*6*_ solvent, excess reagents, and a substantial portion of the unbound heme. NMR samples contained protein concentration of approximately 0.6 mM for ^13^C/^15^N-labeled ZnPPIX H-NOX complex, prepared in 50 mM potassium phosphate buffer pH = 7.0, 30 mM NaCl, and 10% (v/v) D_2_O. The UV–Vis absorption spectrum of the *Ns* H-NOX C139A mutant in complex with ZnPPIX indicates a shift in the Soret-band absorption maximum from 429 nm in the heme-bound form to 421 nm, demonstrating the formation of a new stable complex (ZnPPIX-bound form) (Fig. 2).

### NMR experiments

The selected solvent system for all NMR experiments is 90% H_2_O − 10% D_2_O. 2D ^1^H-^15^N and ^1^H-^13^C HSQC, 3D HNCA, 3D HN(CA)CO, 3D HNCO, 3D CBCA(CO)NH, 3D HNCACB NMR experiments were recorded on a Bruker AVANCE III 950 MHz spectrometer equipped with a cryogenically cooled 5 mm ^1^H/^13^C/^15^N/D Z-gradient probe (TCI). 3D HNHA, 3D HBHA(CO)NH, aliphatic 3D (H)CCH-TOCSY and 3D ^1^H-^15^N NOESY acquired on a Bruker AVANCE III High Definition four-channel 700 MHz NMR spectrometer, equipped with a TCI. The main acquisition parameters for the NMR experiments are listed in Table 1. All experiments are included in the Bruker pulse program library. All NMR spectra used for resonance assignments were acquired at 298 K, processed with Bruker TopSpin software (version 4.3), and analyzed using the CARA program (Keller 2004).

**Table 1:** List of NMR experiments acquired to perform the sequence specific assignment of *Ns* H-NOX C139A mutant in complex with ZnPPIX and their main acquisition parameters

NMR Experiments	Time Domain data size (points)			Spectral width/Carrier frequency (ppm)			NS	Delay time (s)	Field (MHz)
	t1	t2	t3	F1	F2	F3			
^1^H-^15^N HSQC	256	2048		41/118	14/4.7		4	1.2	950
				(^15^N)	(^1^H)				
^1^H-^13^C HSQC	512	2048		160/80	14/4.7		32	1	700
				(^13^C)	(^1^H)				
HNCA	80	48	2048	30/53.2	32/116	14/4.7	16	1.2	950
				(^13^C)	(^15^N)	(^1^H)			
HNCO	64	48	2048	13/173.5	32/116	14/4.7	8	1.2	950
				(^13^C)	(^15^N)	(^1^H)			
HN(CA)CO	64	48	2048	14/173.5	32/116	14/4.7	24	1.2	950
				(^13^C)	(^15^N)	(^1^H)			
HNCACB	96	48	2048	66/43	32/116	14/4.7	24	1.2	950
				(^13^C)	(^15^N)	(^1^H)			
CBCA(CO)NH	96	48	2048	66/43	32/116	14/4.7	16	1.2	950
				(^13^C)	(^15^N)	(^1^H)			
HNHα	48	96	1024	35/117	14/4.7	14/4.7	32	1	700
				(^13^C)	(^15^N)	(^1^H)			
HBHA(CO)NH	112	40	1024	8/4.7	44/117	14/4.7	16	1	700
				(^13^C)	(^15^N)	(^1^H)			
(H)CCH-TOCSY	128	48	1024	80/39	80/39	14/4.7	16	1	700
				(^13^C)	(^15^N)	(^1^H)			
3D ^1^H-^15^N NOESY	256	48	2048	14/4.7	40/117	14/4.7	16	1	700
				(^1^H)	(^15^N)	(^1^H)			

### Extent of assignments and data deposition

**Fig. 3:**
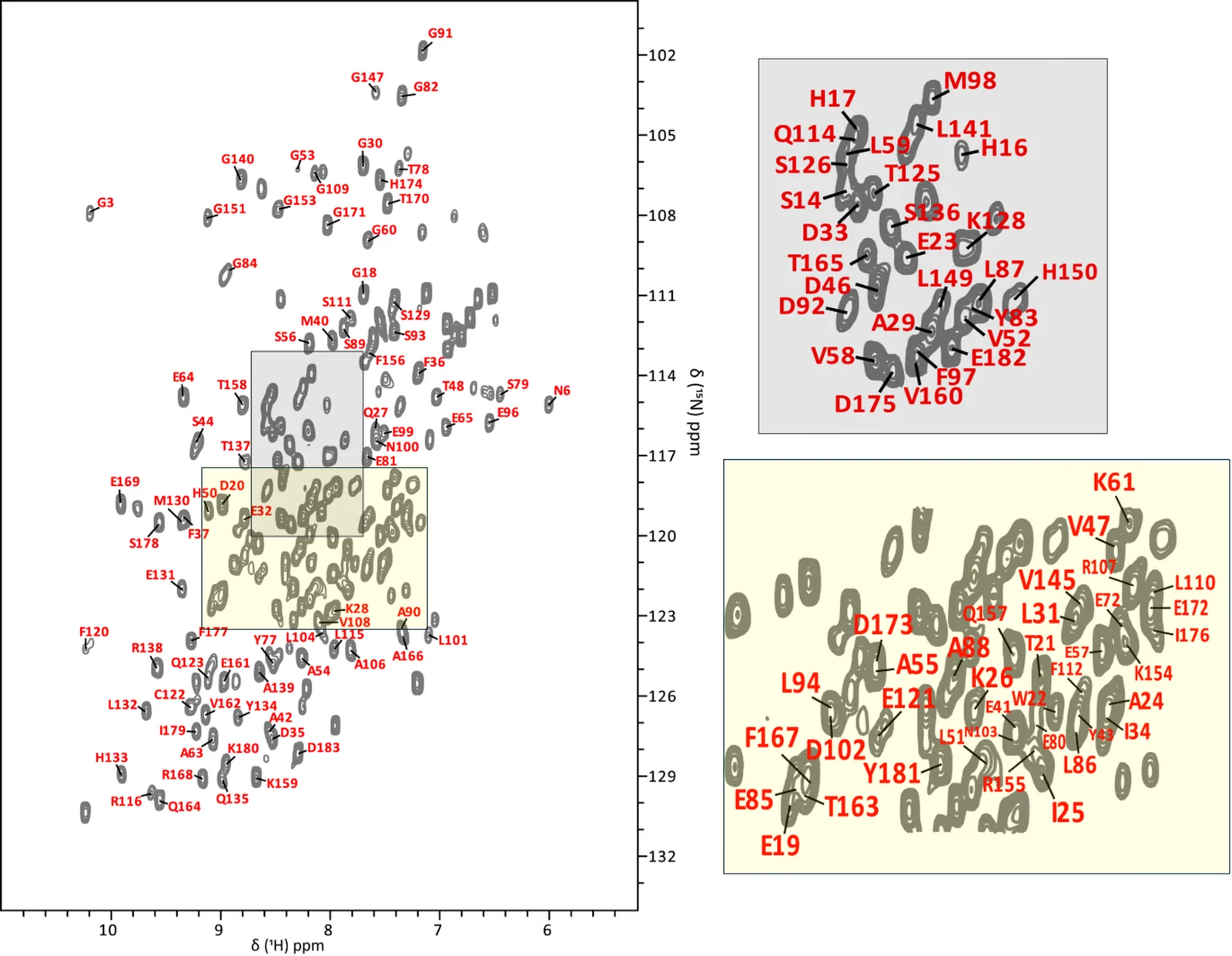
700 MHz 2D ^1^H-^15^N HSQC NMR spectrum of *Ns* H-NOX C139A mutant in complex with ZnPPIX at 298 K. Resonance assignments are labelled in red according to the sequence of the *Ns* H-NOX C139A mutant. The faintly colored highlighted regions of the spectrum (light yellow and grey) present magnified views of the crowded regions of the ^1^H-^15^N HSQC spectrum

**Fig. 4:**
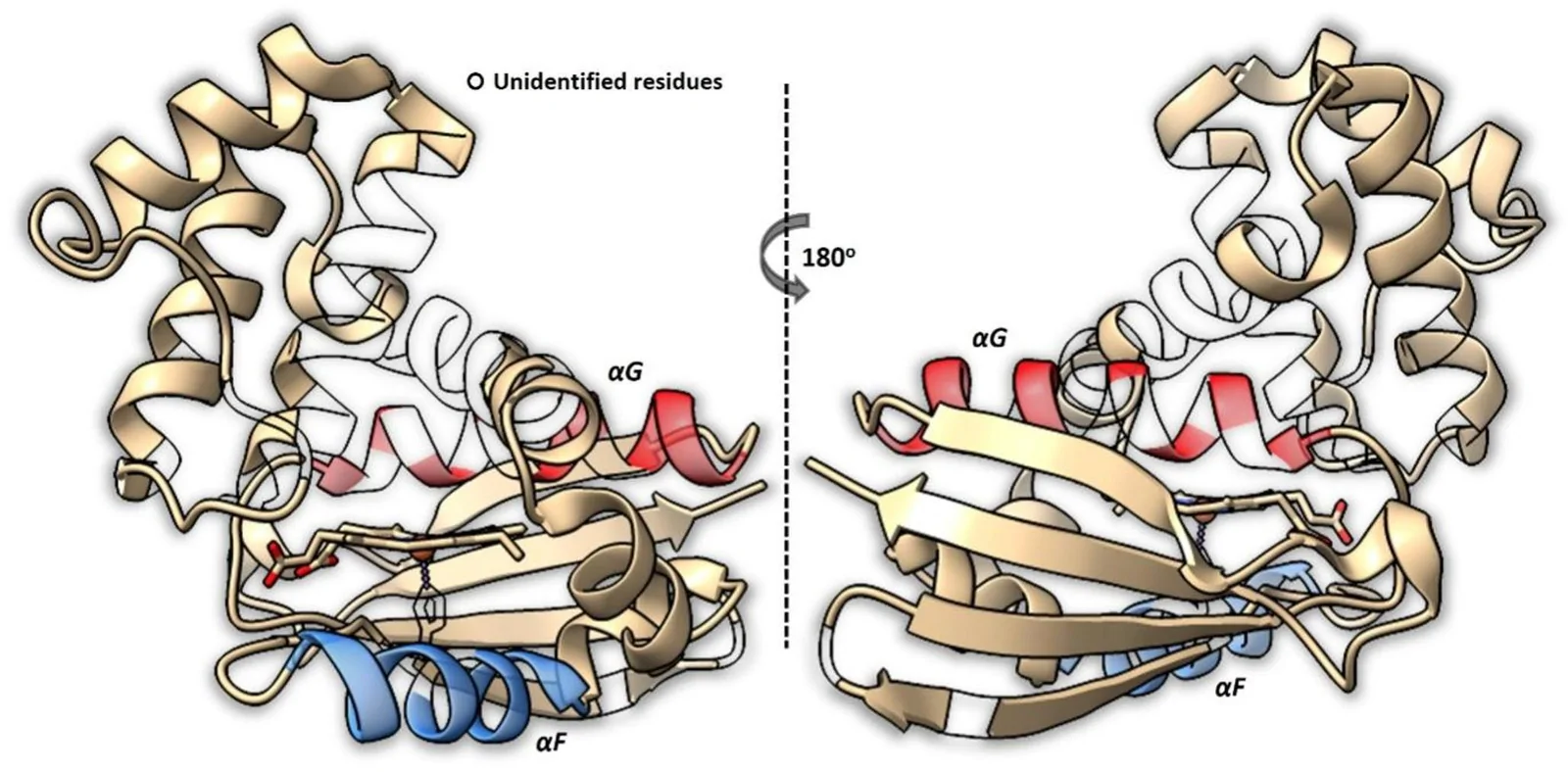
The X-ray crystal structure of Fe(II)-unliganded *Ns* H-NOX (PDB ID: 2O09). The *αF* helix, which according to the crystal structure contains H105 that forms a coordination bond with the heme iron ion, is highlighted in blue. The *αG* helix, which is in the proximal subdomain close to the heme binding pocket, is highlighted in red. The residues that were not identified by NMR spectroscopy in this study are shown uncolored

**Fig. 5:**
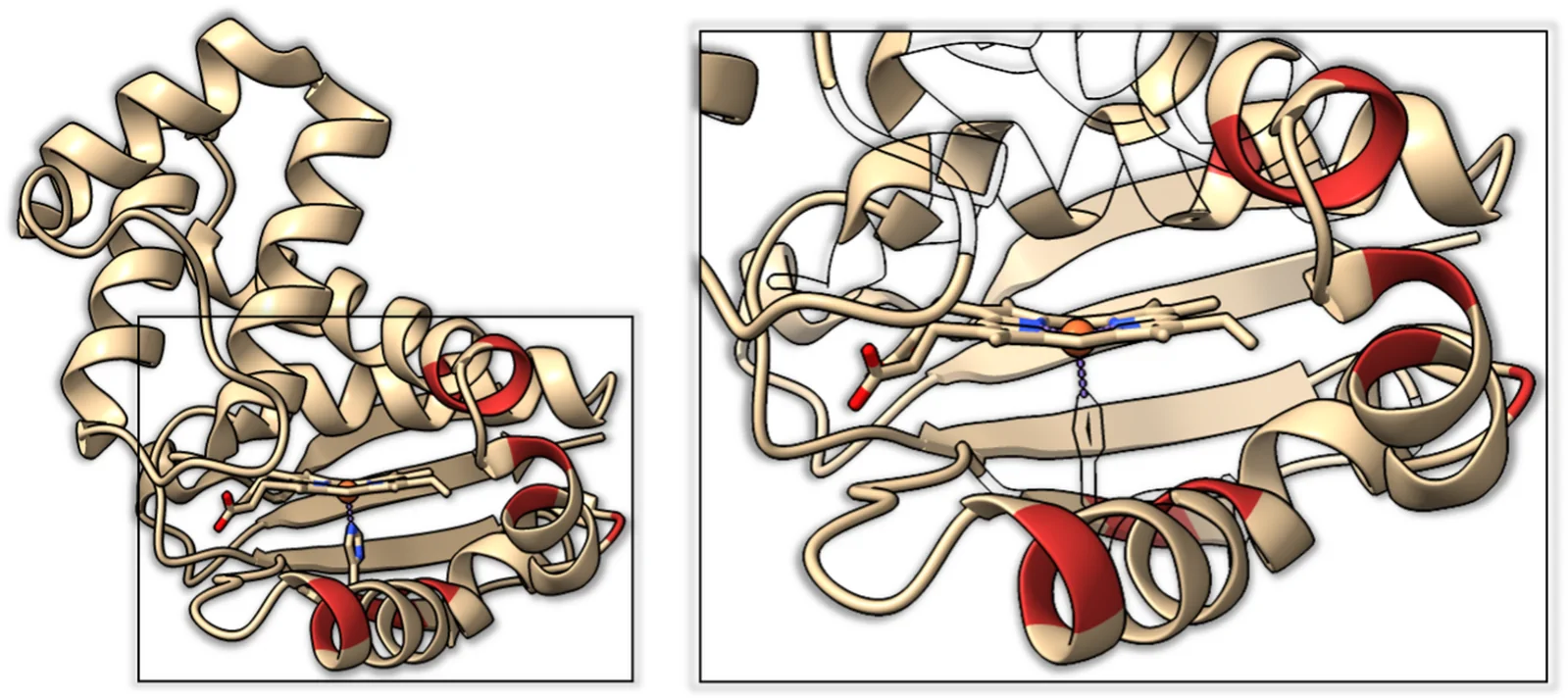
Mapping of residues exhibiting duplicated amide resonances in the *Ns* H-NOX/ZnPPIX complex onto the crystal structure of *Ns* H-NOX (PDB ID: 2O09; left panel). Enlarged view of the heme-binding pocket showing residues with duplicated amide cross-peaks in red, together with pocket-lining residues for which backbone resonance assignments could not be obtained (right panel)

The 1D ^1^H and 2D ^1^H-^15^N HSQC spectra of the *Ns* H-NOX mutant in complex with ZnPPIX are characterized by well-dispersed amide proton correlation peaks and narrow linewidths, indicating a monomeric protein that is well-folded in solution. Combined analysis of two-dimensional and three-dimensional heteronuclear NMR experiments resulted in the sequence-specific assignment of 78% of the backbone ^1^H-^15^N resonances of the protein (Fig. 3), along with 85% of the ^13^C′ (carbonyl carbon), 87% of the ^13^Cα, and 83% of the ^13^Cβ chemical shifts. However, amide proton correlation peaks were not detected for residues M1, Y2, L4, V5, K7, A8, I9, Q10, D11, M12, I13, K15, V38, G39, D45, Y49, L66, L67, I68, A69, F70, G71, Y73, W74, V75, T76, H105, A119, H124, S127, A142, M144, L146, L148, L152 (Fig. 4), nor for the six proline residues (P62, P95, P113, P117, P118 and P143) in the protein sequence. Analysis of the ^1^H-^13^C HSQC, 3D CBCA(CO)NH, 3D HNCACB, 3D HBHA(CO)NH, and 3D (H)CCH-TOCSY spectra enabled the identification of residues P62, P95 and P113, for which the cis/trans conformations were also determined. The ^13^Cβ and ^13^Cγ chemical shifts of proline residues were compared against average reference values reported in the literature (trans-Pro: ^13^Cβ 31.75 ± 0.98, ^13^Cγ 27.26 ± 1.05; cis-Pro: ^13^Cβ 34.16 ± 1.15, ^13^Cγ 24.52 ± 1.09) (Schubert et al. 2002), indicating that all three identified proline residues adopt the trans conformation. The 2D ^1^H–^15^ N HSQC spectrum contains two distinct amide cross-peaks for each of the residues T78, S79, E80, G84, L87, D92, L101, G109, S111, E121, C122, and H150. Nine of these residues (T78, S79, E80, G84, L87, L101, G109, S111, and H150) are in the vicinity of the heme-binding pocket, as indicated by the X-ray crystal structure of *Ns* H-NOX (Fig. 5). The localization of most residues exhibiting duplicated amide resonances around the heme-binding pocket suggests that these amide cross-peaks may reflect alternative local arrangements of the ZnPPIX-bound pocket, potentially involving distinct orientations of ZnPPIX within the binding cavity due to the absence of protein-Zn(II) coordination bond. Additional residues within the heme-binding pocket may also be affected by a different ZnPPIX orientation; however, complete resonance assignment of all residues in this region could not be achieved.

For residues exhibiting duplicated amide resonances, the amide cross-peak displaying the larger peak volume was operationally assigned to the major species in solution. Based on the measured peak volumes, the major and minor species were estimated to account for approximately 60% and 40% of the total population, respectively. The resonance assignments reported in the present study correspond to the major H-NOX/ZnPPIX species. In addition to the duplicated amide resonances described above, 20 further amide cross-peaks are observed in the ^1^H-^15^N HSQC spectrum. These resonances may originate from a minor apo-state (heme-unbound state) population generated during ODQ treatment (Tsai et al. 2010). Oxidation of the native heme iron from Fe(II) to Fe(III) gives rise to a paramagnetic heme-bound species, for which resonances, particularly those arising from residues located in the vicinity of the heme-binding pocket, are expected to undergo pronounced paramagnetic line broadening and hyperfine shifts and may therefore become severely attenuated or undetectable under the experimental conditions. The 20 additional cross-peaks are thus more plausibly attributed to a heme-unbound population formed following oxidation-induced heme dissociation. Supporting this interpretation, a similar pattern of amide resonances has been reproducibly observed upon treatment of native *Ns* H-NOX with ODQ. Loss of the heme cofactor may destabilize the H-NOX fold and increase conformational disorder, potentially giving rise to a partially structured or partially unfolded species that does not efficiently incorporate ZnPPIX. Thus, in addition to the spectral heterogeneity of the H-NOX/ZnPPIX sample, the spectrum may contain contributions from a minor, structurally perturbed apo-state population generated during ODQ-mediated sample preparation.

According to the X-ray crystal structure of *Ns* H-NOX, among the unassigned residues, V38 and G39 are located within loop regions and may be broadened beyond detection due to conformational exchange. Furthermore, residues A142, M144, L146, L148, and L152 are located within helix *αG* (L141-F156) (Fig. 4), in close proximity to the heme-binding pocket in the proximal subdomain (Wittenborn and Marletta 2021), a region that may constitute a structurally and dynamically important element of the *Ns* H-NOX protein and may undergo conformational exchange accompanied by increased local flexibility upon ZnPPIX binding. (Herzik et al. 2014).

The prediction of the secondary structure elements of the *Ns* H-NOX mutant in complex with ZnPPIX was performed using the TALOS-N software. Data extraction from TALOS-N was achieved by providing as input the ^13^C chemical shifts of Cα, Cβ, and CO atoms, the ^15^N chemical shifts of N, HN (amide nitrogen and amide proton), as well as the Hα chemical shifts (Shen and Bax 2015).

**Fig. 6:**
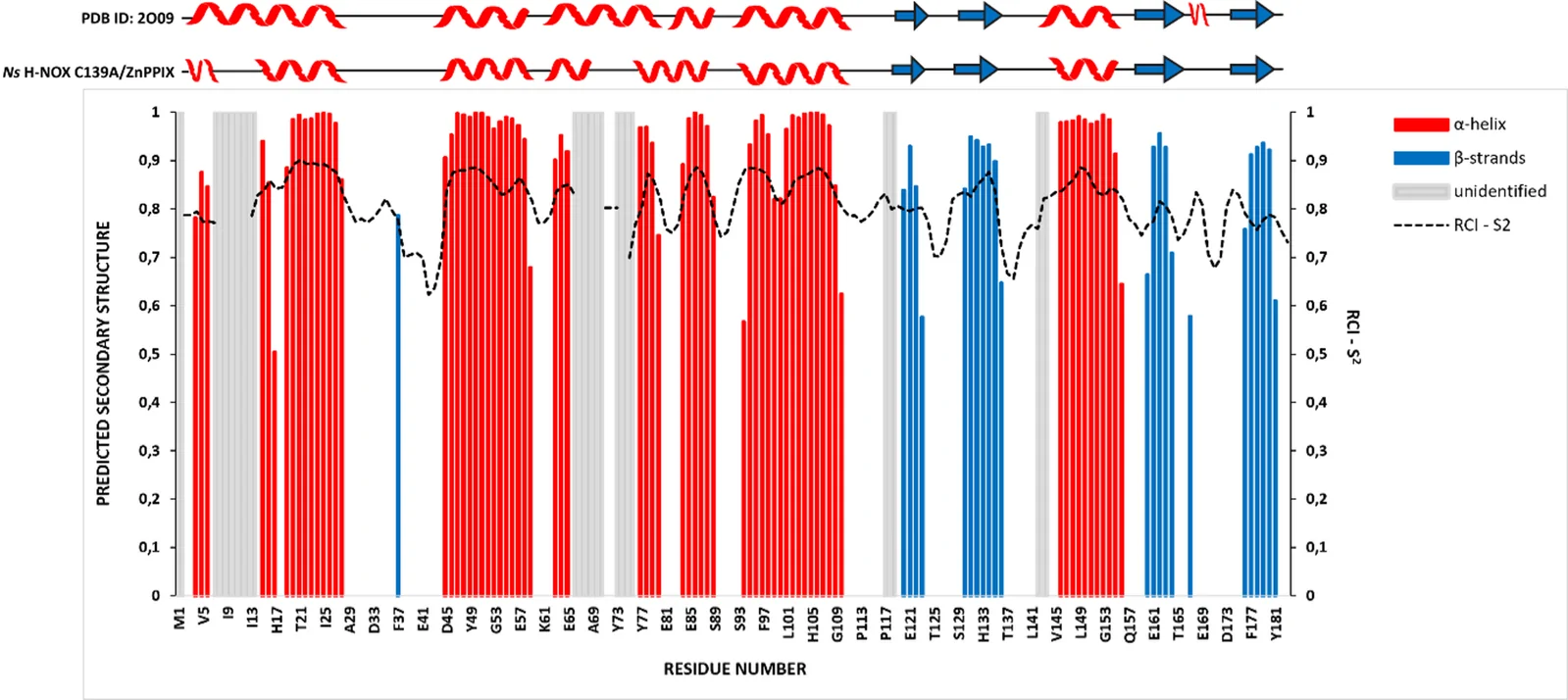
Estimation of the secondary structure of the *Ns* H-NOX C139A mutant in complex with ZnPPIX using the TALOS-N software. The graph displays the predicted secondary structure probabilities (left axis) and the RCI-S^2^ (Random Coil Index-S^2^) values (right axis) for the assigned residues. The color-coded bars denote *β*-strands in blue and *α*-helices in red, whereas the black dashed line represents the RCI-S^2^ values. Light-grey bars indicate unassigned regions of the *Ns* H-NOX sequence. Gaps in the RCI-S² values indicate residues for which TALOS-N could not provide RCI-S² values because of missing chemical shift assignments. For comparison, secondary-structure elements derived from X-ray crystal structures and TALOS-N predictions *(*Shen and Bax 2015*)*are displayed at the top of the plot. For the X-ray structures, the secondary-structure elements were extracted using PDBsum (Laskowski et al. 2018) from PDB entry 2O09 (native *Ns* H-NOX form)

According to the empirical prediction generated by TALOS-N, the *Ns* H-NOX mutant consists of seven *α*-helices and four *β*-strands, adopting a *ααααααββαββ* topology. These data show significant similarity to the secondary structure elements observed in the X-ray crystal structure of *Ns* H-NOX, which exhibits a *αααααββαβαβ* topology (Fig. 6). The apparent reduction in *α*-helical content preceding the first two *β*-strands, as shown in the upper part of the plot in Fig. 6, is attributable to the unassigned regions L4–K15 and L66–T76, for which TALOS-N could not generate secondary structure predictions. Consequently, the corresponding *α*-helical regions appear artificially discontinuous in the plot (Fig. 6). In the presence of the native ligand (heme B), no prediction was obtained for the L101–G109 region due to the lack of assignment. This region, which corresponds to the helix *αF* (N100-F112) based on the X-ray crystal structure of *Ns* H-NOX (PDB ID: 2O09) (Fig. 4), exhibits significant residue conservation among different H-NOX proteins and includes H105. The latter forms a coordination bond with the Fe(II) ion of the heme via the Nε2 nitrogen atom of the histidine imidazole ring (according to the X-ray crystal structure of *Ns* H-NOX, PDB ID: 2O09), thereby establishing a direct link between this *α*-helix and the heme cofactor.

Substitution of heme B with ZnPPIX allows the identification of all residues belonging to helix *αF*. In ZnPPIX, the tetra-coordinated zinc ion in ZnPPIX is unable to form a metal chelating bond with H105, preventing direct coordination between the ZnPPIX and this helix. This substitution likely stabilizes the *αF* helix by limiting cofactor rearrangements within the binding cavity, which normally increases upon NO coordination (Ma et al. 2007). As a result, the *αF* helix maintains a well-defined orientation that facilitates the assignment of its nuclear resonances, while the overall structure of this region remains unaffected.

The NMR resonance assignment of this H-NOX complex is expected to have a significant impact in the study of the enzyme function, since it allows the NMR to experimentally investigate the dynamics of the NO signaling and the activation or inhibition mechanism of a significant pharmaceutical target, such as the sGC enzyme.

## Data Availability

Chemical shift assignments of the *Ns* H-NOX complex with ZnPPIX, reported in this manuscript are deposited in the Biological Magnetic Resonance Data Bank (BMRB) (https://bmrb.io) under the accession number 53820.
